# Supplementary material for: Direct calculation of the ionic mobility in superionic conductors
Source: Sci Rep. 2022 Nov 19;12:19930. doi: 10.1038/s41598-022-21561-1 (PMC9675797; doi:10.1038/s41598-022-21561-1)
Supplement: Supplementary file 1 — Supplementary Information. [file 41598_2022_21561_MOESM1_ESM.pdf]

Direct calculation of the ionic mobility in superionic conductors: Supplementary Information

Alexandra Carvalho[1,2,*], **Suchit Negi**[1,2], **and Antonio H. Castro Neto**[1,2,3]

[1]Institute for Functional Intelligent Materials, National University of Singapore, 117544 Singapore
[2]Centre for Advanced 2D Materials, National University of Singapore, 117546 Singapore
[3]Department of Materials Science Engineering, National University of Singapore, 117575 Singapore
[*]carvalho@nus.edu.sg

S1 Non-linear regime

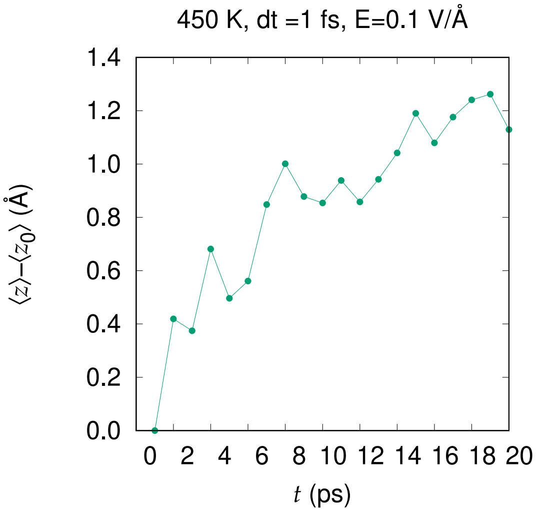

Figure S1. Non-linear effects (saturation) observed for longer integration times, for α-AgI, for an electric field of 0.1 V/Å, at a temperature of 450 K.

For longer integration times, $\langle v \rangle$ is no longer constant. This is due to the accumulation of Ag^+ ions close to $z = L$, and the corresponding screening of the external electric field. Such transient has been observed experimentally in DC measurements[1]. Additionally, if the simulation time extends too long beyond the linear regime, we observe spurious effects, such as Ag ions escaping to the vacuum space, which should be avoided.

S2 Activation energy

An activation energy (E_a) can be obtained from the calculated temperature dependence of the ion mobility in α-AgI (Fig. S2). We obtain E_a=0.032±0.017 eV, which is smaller than the experimental value 0.045 eV[2]-0.050 eV[3]. For α-$RbAg_4I_5$, we obtain E_a=0.026 eV (Fig. S3). This deviates from the experimental value E_a=0.071 eV that we fit from the experimental data in Ref.[4] (using μ vs. T, for direct comparison with our result). Both activation energies are however consistent with the observation of ionic Hall effect, which is only believed to be present when the activation energy for migration is very low.[5]

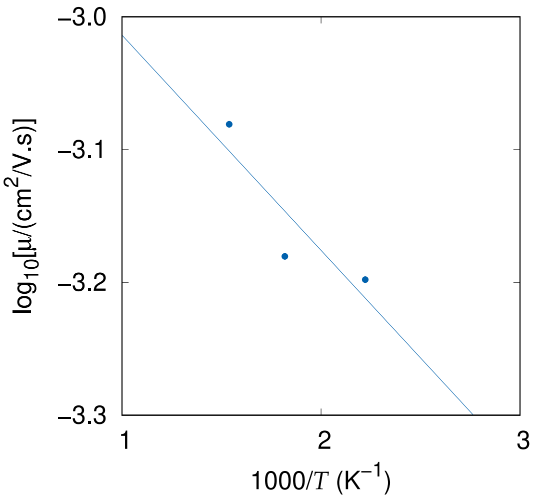

Figure S2. Arrhenius plot for the ionic mobility of α-AgI as a function of the temperature.

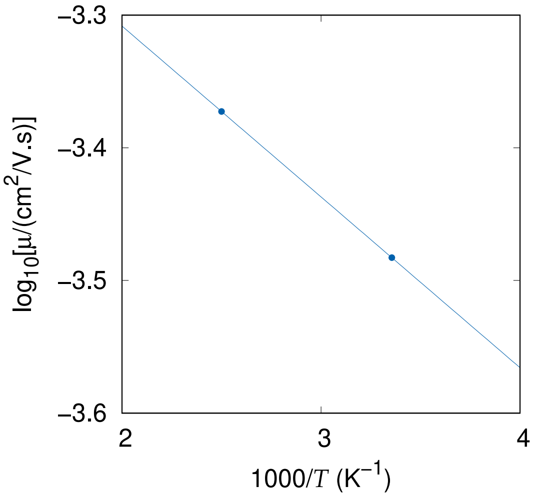

Figure S3. Arrhenius plot for the ionic mobility of α-$RbAg_4I_5$ as a function of the temperature.

S3 Joule effect

In Fig. S4, we show the temperature drift due to Joule effect for a moderate electric field (where $v(E)$ is still in the linear regime). The temperature varies linearly with time despite the drastic departure from the original temperature.

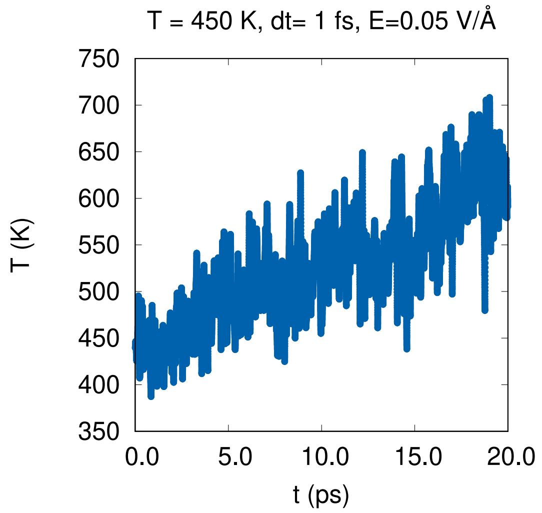

Figure S4. Joule effect-related temperature drift in α-AgI for a longer integration time, for an electric field of 0.05 V/Å, and an initial temperature of 450 K.

References

1. Agrawal, R. C. DC polarisation: An experimental tool in the study of ionic conductors. *Indian J. Pure & Appl. Phys.* **37**, 294–301 (1999).
2. Hassan, M., Al-Hakimi, A. N. *et al.* Electrical conductivity of AgI–CdI_2–KI and AgI–CuI–KI ionic conducting systems. *Arab. J. Chem.* **4**, 45–49 (2011).
3. Funke, K. AgI-type solid electrolytes. *Prog. solid state chemistry* **11**, 345–402 (1976).
4. Stuhrmann, C., Kreiterling, H. & Funke, K. Ionic hall effect measured in rubidium silver iodide. *Solid state ionics* **154**, 109–112 (2002).
5. Slifkin, L. The ionic Hall effect in crystals. In Laskar, A., Boucquet, J. L., Brebec, G. & Monty, C. (eds.) *Diffusion in Materials*, 1–17 (Kluwer Academic Publishers, Dordrecht, 1990).
